# Emotion regulation is the ability to exert control over one’s own emotional state

**DOI:** 10.1192/j.eurpsy.2023.1211

**Published:** 2023-07-19

**Authors:** R. Hernandez Anton, J. P. D. L. V. García, J. O. B. González, R. V. Casal

**Affiliations:** 1 Consorci Sanitari del Maresme, Barcelona; 2 Consorci Sanitari del Maresme, Barcelona, Spain

## Abstract

**Introduction:**

This program project arises from the consternation of psychotherapists at the increase in self-injurious behaviors in the child and adolescent population.

Currently, in consultation, we are seeing many cases that do not match the conditions (anxiety disorders, depression, attention deficit disorder and hyperactivity…) described by current classifications. These cases have a common feature: emotional dysregulation.

**Objectives:**

- Reduce the discomfort and emotional pain of the patient.- Increase the patient’s skills.

- Improve patient motivation.

- Generalize to the natural environment.

- Structure the environment.

**Methods:**

The program is directed to peoople between the ages of 12 and 18. They are divided into two groups: one from 12 to 15 years old and another from 16 to 18 years old.

The groups are a maximum of 8 adolescents. Parents also participate.

These are closed groups.

The duration of each session is one hour or one hour and a half.

A therapeutic contract is signed.

**Results:**

We will use different scales to measure the evolution of the patients. The following scales will be passed at the beginning and at the end: DERS, EGD, DASS 21, GHQ-12.

Dialectical dilemmas in families will be worked on. These results will be collected and compared with those at the end of the program.

**Conclusions:**

People with emotional dysregulation sometimes do not have the necessary skills to regulate emotions. With this program, we intend to carry out training in skills (mindfulness; middle path; tolerance to discomfort; emotional regulation), structured in modules, in addition to relying on individual therapy.

Given this increase in deregulated children and young people, we see ourselves in the need to train ourselves and address these cases from a different point of view.

**Disclosure of Interest:**

None Declared

